# Dual-Functional Phosphorene Nanocomposite Membranes for the Treatment of Perfluorinated Water: An Investigation of Perfluorooctanoic Acid Removal via Filtration Combined with Ultraviolet Irradiation or Oxygenation

**DOI:** 10.3390/membranes11010018

**Published:** 2020-12-25

**Authors:** Joyner Eke, Lillian Banks, M. Abdul Mottaleb, Andrew J. Morris, Olga V. Tsyusko, Isabel C. Escobar

**Affiliations:** 1 Center of Membrane Sciences, Department of Chemical and Materials Engineering, University of Kentucky, 177 FPAT, Lexington, KY 40506-0046, USA; joyner.eke@uky.edu (J.E.); Lillian.Banks@uky.edu (L.B.); 2 College of Medicine, University of Kentucky, 177 FPAT, Lexington, KY 40506-0046, USA; mabdul.mottaleb@slu.edu (M.A.M.); a.j.morris@uky.edu (A.J.M.); 3 Institute of Drug & Biotherapeutic Innovation, Saint Louis University, 1100 South Grand Blvd, Saint Louis, MO 63104, USA; 4 Department of Plant and Soil Sciences, University of Kentucky, 1100 S. Limestone St., Lexington, KY 40546-0091, USA; olga.tsyusko@uky.edu

**Keywords:** 2-dimensional materials, nanofiltration, per- and polyfluorinated compounds (PFAS), functionalization

## Abstract

Nanomaterials with tunable properties show promise because of their size-dependent electronic structure and controllable physical properties. The purpose of this research was to develop and validate environmentally safe nanomaterial-based approach for treatment of drinking water including removal and degradation of per- and polyfluorinated chemicals (PFAS). PFAS are surfactant chemicals with broad uses that are now recognized as contaminants with a significant risk to human health. They are commonly used in household and industrial products. They are extremely persistent in the environment because they possess both hydrophobic fluorine-saturated carbon chains and hydrophilic functional groups, along with being oleophobic. Traditional drinking water treatment technologies are usually ineffective for the removal of PFAS from contaminated waters, because they are normally present in exiguous concentrations and have unique properties that make them persistent. Therefore, there is a critical need for safe and efficient remediation methods for PFAS, particularly in drinking water. The proposed novel approach has also a potential application for decreasing PFAS background levels in analytical systems. In this study, nanocomposite membranes composed of sulfonated poly ether ether ketone (SPEEK) and two-dimensional phosphorene were fabricated, and they obtained on average 99% rejection of perfluorooctanoic acid (PFOA) alongside with a 99% removal from the PFOA that accumulated on surface of the membrane. The removal of PFOA accumulated on the membrane surface achieved 99% after the membranes were treated with ultraviolet (UV) photolysis and liquid aerobic oxidation.

## 1. Introduction

Based upon the success of graphene, two dimensional (2D) materials have excited scientists worldwide. As a result, much research has been tailored towards developing the next generation of materials that may be able to overcome one of the main limitations of graphene, which is the absence of a band gap [1]. Phosphorus which constitutes approximately 0.1% of the Earth’s crust is one of the most abundant elements [2], and it exists as several allotropes. White and red phosphorus are the most commonly seen allotropes used typically for making explosives and safety matches [3]. Black phosphorus (BP), though rarely mentioned, is the most stable allotrope of phosphorus [4], and it combines high carrier mobility with a fundamental band gap [5]. Graphite and black phosphorus (BP) are the only known monotypic van der Waals crystals [6,7]. Unlike carbon, phosphorus has only three valance electrons which leads to BP being semiconducting, since each atom is bonded to three neighboring atoms [6]. Exfoliated, p-type semiconducting BP flakes possess mobilities of ~200–1000 cm^2^/V-s at room temperature, current on/off ratios of ~104 and anisotropic transport. Consequently, BP shows promise as a nanomaterial that could complement or exceed the electronic, spintronic, and optoelectronic properties of graphene [8,9]. Phosphorene is the single atomic layer of BP that shows semiconducting properties [10]. Phosphorene distinguishes itself from other 2D layered materials by its unique structural characteristics, relatively large direct band gap and good charge carrier mobilities [11].

Photocatalysts absorb photons to increase the chemical rate of reaction [12]. Reactions are activated by the absorption of a photon with sufficient energy (equivalent to or greater than the band-gap energy of the catalyst). The photon absorption leads to a charge separation due to elevation of an electron (e^−^) from the valence band of the semiconductor catalyst to the conduction band [13]. Phosphorene exhibits characteristics that are desirable for photocatalytic applications, which include quantum confinement in the direction perpendicular to the 2D plane signifying optical properties, large lateral size with a high specific surface area and ratio of exposed surface atoms, high absorbance and strong interaction with light [14,15,16,17]. Furthermore, phosphorene is a direct and narrow band gap semiconductor, thus, it could efficiently harvest low energy photons during photocatalysis, which can be tuned appropriately for photon absorption in the ultraviolet, visible light and the near-infrared region of the solar spectrum. Therefore, these properties of phosphorene have the potential to be explored in designing low fouling surfaces, such as membranes for contaminant removal. Recently, phosphorene has been used as an additive to produce stable nanohybrid membranes highly selective for molecules and ions [18].

Perfluoroalkyl substances (PFAS) are a group of man-made surfactant chemicals that were first produced in the 1940s. PFAS can be found in many consumer products including food packaging, household cleaners and fire-fighting foams. PFAS have been a concern because they do not degrade and are very persistent in the environment [19]. PFAS are organic fluorochemicals where at least one carbon-hydrogen bond on the hydrocarbon chain is replaced by a fluorine–carbon bond. Fluorine is one the most reactive elements when not bonded, but when it has been bonded, it is extremely stable. Fluorinated hydrocarbons are resistant to high temperatures, strong acids and bases and are nonflammable as well [20]. This stability of PFAS makes it virtually nondegradable and allows for PFAS to build up in the environment, in marine animals and mammals, including humans. There have been several studies performed that show evidence of PFAS having adverse effects on human health because of their environmental persistence and widespread human exposure and toxicity [21,22,23]. They are extremely persistent in the environment because they possess both hydrophobic fluorine-saturated carbon chains and hydrophilic functional groups, along with being oleophobic [24]. PFAS have been shown to have carcinogenic properties as well as developmental toxicity [20,25]. As the chain length of the compounds increases so does the toxicity of their effects [26]. In 2009, the EPA labeled two PFAS substances, perfluorooctanoic acid (PFOA) and perfluorooctane sulfonate (PFOS), as contaminants of potential concern in drinking water [27], and it has set a lifetime health advisory at 70 ng/L for PFAS in drinking water [28].

PFOA and PFOS are two of the most common PFAS substances produced and their production has now been banned in most of Europe and the United States. The degradation of PFOA, leads to the formation of two intermediate products, which are less fluorinated carboxylic acids and shorter-chain PFASs. The presence of the carboxylic acids indicates the cleavage of C−F bonds and H/F exchange, while formation of short chain PFASs implicates the scission of C−C bonds [29]. The treatment technologies currently available for the removal and/or degradation of PFAS compounds are limited to adsorption, advanced photochemical oxidation, sonochemical decomposition, filtration, and air-sparged hydrocyclone technology [30]. For adsorption, granular activated carbon in the absence of organic matter is effective for the removal of long chain perfluoroalkyl acid but it is ineffective against short chain perfluoroalkyl acids [31]. Sonolysis involves the use of ultrasound waves to create cavitation. During the process of cavitation, bubbles collapse and adiabatically generate high pressure and temperature conditions that pyrolyze perfluorinated compounds [32]. Sonolysis has been observed to breakdown PFAS compounds on the laboratory scale, but it has not been commercialized because of design challenges during the cavitation propagation [31]. Advanced oxidation processes that have successfully degraded PFAS include ultraviolet (UV) irradiation and electrochemical techniques [33]. Photochemical oxidation is an indirect photolysis technique, which degrades contaminants by reacting with reactive radicals. Adding a photocatalyst to UV photolysis of PFAS enhanced the ability of the process to degrade the material [34]. Catalysts such as TiO^2^, Fe^3+^, S_2_O_8_^2−^, IO^4−^, and CO_3_^2−^, in combination with UV, efficiently degraded PFAS owing to formation of reactive and potent oxidative species such as CO^3−^•, H•, OH•, and PFAS complexes [35]. Direct photolysis of PFASs tends to have relatively low removal efficiencies and fluoride yields compared with other processes and thus needs additional processes to reach complete degradation [34].

Oxygen is a cheap, abundant and green oxidant, which usually generates water as the only stoichiometric byproduct, and recent research efforts have been tailored towards the development of liquid phase aerobic oxidation methods to combat the negative impact of the inorganic oxidants, like potassium permanganate, chromium trioxide, and manganese dioxide [36]. Under ambient conditions, oxygen in its ground state is unreactive with organic molecules; hence, a catalyst is often necessary to control the selective oxidation of a molecule [37]. Palladium catalyzed aerobic oxidations are the most studied and have been successful in the conversion of alcohols to ketones and aldehydes [38].

Recently, a temperature-responsive membrane composed of poly-N-isopropylacrylamide (PNIPAAm) on polyvinylidene difluoride (PVDF) membranes acted as polymeric adsorber to remove PFOA successfully [39]; however, PFOA was not destroyed. Therefore, there is a critical need for safe and efficient remediation methods for PFAS, particularly in drinking water.

**Figure 1:**
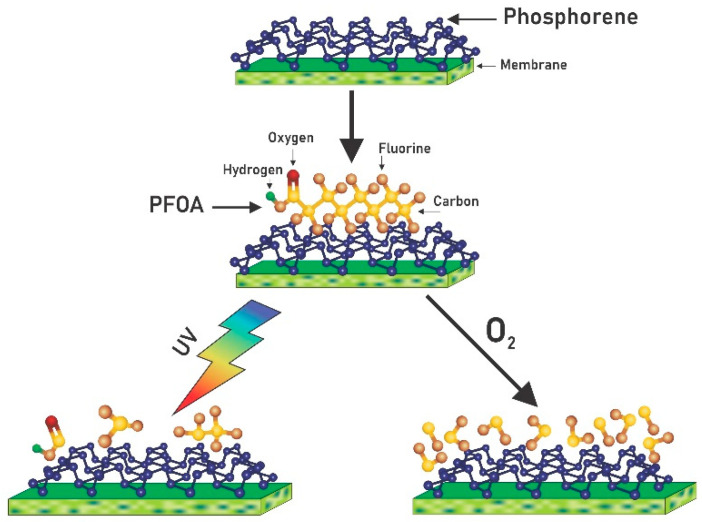
Schematics of experimental process showing the filtration of perfluorooctanoic acid (PFOA) on phosphorene membranes followed by either treatment using UV irradiation or liquid aerobic oxidation.

Nanocomposite membranes with tunable properties show promise for numerous technologies [40,41,42,43] because of their size-dependent electronic structure and controllable physical properties. The purpose of this research is to develop and validate environmentally safe nanomaterial-based approaches for treatment of drinking water including degradation of per- and polyfluorinated chemicals (PFAS). Specifically relevant to the field of liquid separations using membranes, the band gap of phosphorene provides electronic [44] and photocatalytic [17] properties, which are proposed to make reactive membranes to simultaneously remove and destroy PFAS. With the high toxicity and corrosive issues encountered with metal-based photocatalysts (oxides, sulfides, and nitrides of titanium, tungsten, cadmium, and transition-metal dichalcogenides), phosphorene can act as a metal-free photocatalyst to degrade organic compounds in the feed solution to make reactive membranes. In this study, charged nanofiltration membranes were synthesized by blending polysulfone (PSf) with sulfonated poly (ether ether ketone) with phosphorene nanoparticles. The goal of this study was to assess the potential of phosphorene membranes for contaminant removal. Here, a nanohybrid nanofiltration (NF) membrane with tailored selectivity for the removal of PFOA was used. After filtration, the removal and/or destruction of the PFOA that accumulated on the surface of the membranes was investigated using UV and oxygenation treatments. Figure 1 shows a schematic of the experimental process.

## 2. Materials and Methods

### 2.1. Materials

Perfluorooctanoic acid (PFOA) was purchased from Sigma Aldrich (St Louis, MO, USA). For the sulfonation reaction, polysulfone (PSf), N-methyl pyrrolidone (NMP), poly ether ether ketone (PEEK), and concentrated sulfuric acid were purchased from VWR (Radnor, PA, USA). Black phosphorus used for the synthesis of phosphorene was purchased from Smart Elements (Vienna, Austria).

### 2.2. Preparation of Phosphorene

Phosphorene was synthesized by chemically exfoliating black phosphorus according to previously described techniques [45,46]. In summary, NMP and NaOH with a volume ratio of 1:1 were mixed and degassed on an ultrasonicator (P70H, Elma Elmasonic P, Singen, Germany) for five minutes. Then, 100 mg of black phosphorus was introduced into the mixture and sonicated for five hours at frequency and power of 37 KHz and 80%, respectively. The temperature of 30 °C was maintained throughout the experiment. This was then followed by centrifugation at 4000 rpm for 23 min. After centrifugation, the supernatant was collected and used for the experiment.

### 2.3. Sulfonation of Poly Ether Ether Ketone

Poly ether ether ketone was sulfonated as previously described [46]. Briefly, PEEK pellets were oven dried at 60 °C overnight, after drying the pellets were dissolved in concentrated sulfuric acid solution (98%) for three days until a homogenous solution was formed at 25 °C. The solution was gradually added into an ice water bath which was vigorously stirred to precipitate SPEEK (sulfonated poly ether ether ketone) polymer. SPEEK was washed in deionized water until a pH of 7 was achieved. Then it was oven dried at 60 °C and stored for usage.

### 2.4. Water Flux Analysis

The membrane used in this study was a polymeric blend of polysulfone and SPEEK, the process has been described here [18]. The dope solution was a (95/5%) ratio of PSf and SPEEK, and 0.5 volume wt.% of exfoliated phosphorene in basic NMP. Dead-end filtration studies were performed using a 50-mL Amicon stirred cell (model 8010–50 mL, Millipore Sigma, Burlington, MA, USA) under continuous stirring in a batch mode. A Whatman filter paper (110 mm) was used as a support for the membranes during the experiment. The filtration was done under a constant pressure of 2.06 bar at room temperature. The time for 2 mL of water to pass through membranes with an area of 13.4 cm^2^ was recorded, and the water flux, J (LMH), was calculated using Equation (1)

(1) $$J\;=\;\frac{V}{A\Delta t}$$

where *V* is the volume of solution through the membrane in L, and *A* is the active filtration area of the membrane cell in m^2^, and t is the permeation time.

### 2.5. Determination of the Water Interaction Parameter of the Membranes

A drop shape analyzer (Kruss DSA100, Matthews, NC, USA) was used to determine the wettability or water interaction parameter of the membrane by estimating the contact angle between water and the solid surface of the membrane. The wettability of a membrane surface plays a key role in water permeability and organic material adsorption [47]. For a liquid drop on a flat horizontal surface, the contact angle can be described as the tangential angle formed at the point of contact of the liquid on the solid surface. It denotes the equilibrium point of all surface tension forces acting on the boundary layer at the point of contact [47]. A contact angle value lower than 90° typically implies hydrophilicity of the material and values greater than 90° usually denotes hydrophobicity.

### 2.6. Treatment Processes

After filtration of PFOA through the membrane, the membranes were removed and treated using two methods, as shown in Figure 1. The first was a photolysis system consisting of UV irradiation of the catalytic phosphorene membrane, while the second was a liquid aerobic oxidation system consisting of oxygenating the catalytic phosphorene membrane. For the first setup, ultraviolet irradiation was supplied with a UV lamp (Spectroline Model EA-160, Westbury, NY, USA) at 365 nm. The membranes were exposed for 200 min and experiments were performed in the dark. For the second setup, oxygen was bubbled at a constant flowrate of 3 L/min onto the surface of the membrane for 280 min, experiments were performed under visible light. These time durations were chosen based on previous trial experiments. A series of tests were conducted and after 120 min, significant removal of PFOA had not happened, hence it was decided to increase experimental time. After treatment, the membranes were cleaned by reverse flow filtration, and the permeates from the backwash process were tested for PFOA. Each experiment was replicated three times, and the average concentrations of PFOA in the permeate and membrane surface were used in the calculation of adsorbed PFOA removal. Equation (2) was used to determine the rejection of PFOA in the permeates.

R = (1 − (C_pr_/C_s_)) × 100% (2)

where C_pr_ is the PFOA concentration in the permeate after reverse flow filtration, and C_s_ is the concentration on the membrane surface, which is calculated from the difference between initial PFOA feed concentration (C_f_) and concentration of PFOA in the permeate after filtration (C_P_).

### 2.7. Membrane PFOA Adsorption Analysis

To study PFOA adsorption control performance of the membranes, PFOA solution at a concentration of 100 mg/L was filtered. First, it is important to note that PFOA is not a typical foulant and other studies have not observed any significant irreversible fouling on NF 270 membranes due to PFOA presence [48]. For purposes of this study, the observed accumulation of PFOA on the external surface or within the pores of the membrane and at the pore walls is addressed as adsorption. A high PFOA concentration of 100 mg/L was used for filtration to examine not only the removal efficiency of phosphorene modified membranes but also to study the removal of the PFOA adsorbed to the membrane surfaces under UV irradiation and oxygenation treatments. The PFOA-adsorbed membranes after filtration were subjected to two treatment methods: Photolysis by irradiation with ultraviolet light (Spectroline Model EA-160, Westbury, NY, USA) at a wavelength of 365 nm and catalytic oxygenation by bubbling oxygen on the surface of the membranes in water after filtration. This was followed by reverse-flow filtration of deionized water to eliminate reversibly-adsorbed PFOA from the treatment steps and determine flux recovery of the membrane. The flux of the PFOA solution *J_p_* (LMH) and the flux of the cleaned membrane, *J_r_* were measured at 2.06 bar. The flux recovery (FR) was estimated using Equation (3) [49]. The higher the flux recovery, and the lower the total adsorption ratio, the higher the anti-adsorption property of the membrane [50].

(3) $$\mathrm{FR}\;\left(\%\right)\;=\left(\;\frac{J_{r}}{J}\right)\times \;100$$

The adsorption resistance of the membrane was evaluated using Equations (4)–(6) [49], where R_t_, R_r_, and R_ir_ represent the total adsorption ratio (which indicates the total flux loss from PFOA adsorption), reversible adsorption and irreversible adsorption respectively.

(4) $$R_{t}\;=\;\left(1\;-\;\frac{J_{p}}{J}\right)\times 100$$

(5) $$R_{r}\;=\;\left(\frac{J_{r}-J_{p}}{J}\right)\times \;100$$

(6) $$R_{\mathrm{ir}}\;=\left(\frac{J-J_{r}}{J}\right)\times 100$$

The PFOA rejection of the membrane after filtration and after during reverse flow filtration after each treatment method was determined using Equation (2).

The concentration of PFOA was determined using an liquid chromatography-tandem mass spectrometry (LC-MS/MS) method for per-/polyfluoroalkyl substances from water, according to the one used by Saad et al. [39,51]. Essentially, we employed our previously developed and reported the LC-MS/MS method for per-/polyfluoroalkyl substance (PFAS) analysis. Briefly, PFOA was measured by ultra-performance liquid chromatography (UPLC) coupled electrospray ionization tandem mass spectrometry. A bench top binary Shimadzu chromatograph (Model: LC-20 AD) and SIL 20 AC autosampler interfaced with an AB SCIEX mass spectrometer (MS/MS) (Model: 4000 Q TRAP) were used. In this study, since PFOA was target analyte, mass labeled perfluoro-n-[1,2,3,4-^13^C_4_] octanoic acid as surrogate standard (SS), and mass labeled perfluoro-n-[1,2,3,4-^13^C_4_] heptanoic acid as internal standard (IS) were used. Filtered and diluted water samples (1.0 mL) were prepared containing 40 ng/L SS and 20 ng/L IS. The SS spiked samples, continuous calibration verification (CCV), reagent blank and IS-blank were used as quality controls (QC). Target analyte concentrations and QC performance of the method were determined using IS based calibration curves. A gradient elution of mobile phase containing 20 mM ammonium acetate in pure water (A) and pure methanol (B) was used with a Macherey Nagel analytical column EC 125/2 NUCLEODUR C18 gravity packed with 5 µm particle (length 125 × 2 mm ID) at a constant flow rate of 0.4 mL/min. A 13.51 min gradient with composition of B was started 40% at 0.01 min, 65% at 1 min, 90% at 6 min, 95% at 11.5 min, 40% at 13.51 min with 2 min equilibration time. A volume of 5 µL of standard or samples was injected. Data were collected in negative multiple reaction monitoring (MRM) mode with monitoring of quantitation and qualifier ions for PFOA, SS, and IS. Data acquisition and process were performed using AB Sciex Analyst version 1.4.2 and Multiquant version 3.0 software, respectively. The precursor and product ions monitored were PFOA 412.912 > 368.7, 168.7 *m/z*; SS 416.946 > 371.9 171.7 *m/z*; IS 366.897 > 321.7, 171.6 *m/z* were obtained. Bold face indicates the quantitation ions. The PFOA, SS and IS were eluted from column at retention times of 6.57, 6.58, 6.03 min, respectively. Average spiked SS recovery was for 99.2% and average analyte CCV recoveries 105.4%. Limit of detections (LOD) for target analytes were 0.25 ng/mL at S/N = 4. Seven calibration points with linear dynamic range (LDR) were 1.0–160 ng/mL with R^2^ values of 0.9986. MS was operated with curtain gas 30 psi, negative ESI 4500-volt, temperature 300 °C, and ion sources gas (GS1/GS2) 30 psi.

### 2.8. Measuring Ion Fluoride Concentration

The release of ion fluoride has previously been used when detecting PFOA mineralization [52]. The ion fluoride concentration was measured using Ion Chromatography System (ICS) (Dionex, ICS 3000, Sunnyvale, CA, USA) following the EPA method 300.1 for determination of inorganic ions by IC [53]. Briefly, we used a Dionex AS19 column, 39 mM KOH as the mobile phase with a flow rate of 1 mL/min, and 20 µL for sample injection. NIST traceable Fluoride standards were used with stock standards at 10 mg/L.

### 2.9. Structural and Profile Studies with Scanning Electron Microscope (SEM) and X-ray Photoelectron Spectroscopy (XPS)

SEM studies were carried out to examine the surface characteristics of the membrane after PFOA filtration. The membranes were first submerged and broken in liquid nitrogen to achieve a fractured surface with negligible deformation (stretching and tearing) of the polymeric membranes. Surface images were obtained with the resulting fracture in a scanning electron microscope (SEM), Quanta FEG 250, FEI (ThermoFisher Scientific, Waltham, MA, USA) without conductive coating.

The atomic profile compositions of the membranes were determined using a using a Thermo Scientific (Waltham, MA, USA) K-Alpha XPS apparatus equipped with an Al K (1486.6 eV) source (pass energy of 20 eV). Scans were conducted on the surface and a depth profile study was done on different regions to quantify the presence of fluorine bon in the membranes after each treatment method because it is the only element unique to PFOA in the experimental setup. Depth profiling was performed using an ion beam to etch layers of membrane surfaces and elemental composition was measured after each etching cycle. XPS characterization of phosphorene membranes was performed. Phosphorus, carbon, oxygen, sulfur, and fluorine peaks were fitted using Thermo Scientific™ Avantage Software. The emission current of the X-ray source was 12 mA while the acceleration voltage was 10 kV. The spectra measurement was performed at an emission angle of 90°. The electron energy analyzer operated in FAT mode (Fixed Analyzer Transmission), with a pass energy of for survey scans and high-resolution scans of 50 eV and 20 eV, respectively. The total resolution of this XPS was about 1.1 eV.

### 2.10. Surface Roughness Characterization

For nanofiltration membranes, factors that affect the extent of adsorption of materials on the membrane include membrane surface roughness, surface charge and surface hydrophobicity [54]. The surface roughness of the membranes was measured after reverse flow filtration to determine the effect of PFOA adsorption on the roughness of the membrane. An atomic force microscope (AFM) (Bruker Dimension Icon, Santa Barbara, CA, USA) was used to measure surface roughness. A membrane area of 20 × 20 µm was chosen. Data were collected under tapping mode and evaluated by the average root–mean–squared (RMS) roughness.

### 2.11. Data Analysis

All experiments were replicated three times and the data presented are the averages and standard deviations of the replicates. For statistical analysis, using SPSS software, one-way ANOVA followed by post hoc Tukey’s test for multiple comparisons were performed. These statistical analyses were used to detect significant differences in contact angle and the percentage of PFOA from the membrane under UV or oxygenation treatment under different duration. Significance was determined at α ≤ 0.05. Error bars in the figures represent ± one standard deviation obtained from all experiments being performed in triplicates.

## 3. Results and Discussion

In previous studies, phosphorene membranes were synthesized to characterize the evolution of the polymeric membrane fabrication upon addition to phosphorene [18]. Characterization presented there is summarized here. First, a degree of sulfonation of 0.77 verified that the membranes would not solubilize during filtration and further supported the recipe used here. The average pore diameter at the maximum pore distribution, i.e., the most prevalent pore size, of the SPEEK membranes was 0.022 microns (with smallest and largest detected pores being 0.017 and 0.086 microns), while that of the phosphorene membranes averaged 0.0024 microns (with smallest and largest detected pores being 0.0022 and 0.0078 microns). This indicated that the added phosphorene accumulated within the pores. SPEEK membranes displayed an average hydrophilicity as measured by contact angle of 48.3° ± 0.67°, while phosphorene-membranes had an average contact angle of 81.5° ± 0.64°. This shows that unmodified membranes were more hydrophilic, while phosphorene membranes had a more hydrophobic nature that is associated with the presence of the more hydrophobic phosphorene. It was also observed that both SPEEK membranes and phosphorene membranes were negatively charged in both acidic and basic mediums. At a pH of approximately 6, the zeta potential of SPEEK was −61 ± 4.6 mV while that of the phosphorene membrane was −44 ± 7 mV, which was possibly due to the phosphorene nanoparticles masking some of the sulfonic sites (the source of the negative charge of the membranes). Depth-profile scans found that phosphorus was present throughout the membrane matrix, indicating that phosphorene was present also within the pores of the membranes. In leaching studies, it was observed that phosphorene loss was less than 1% of the initial amount of phosphorene added, implying stability within the membrane matrix. Surface morphology studies indicated that phosphorene membranes had rougher surfaces, while the SPEEK membranes had smoother surfaces, which was likely due to some agglomeration caused by water being used as the nonsolvent during membrane fabrication via NIPS. The membranes modified with phosphorene displayed a higher protein rejection, but lower flux values and flux recovery after filtration possibly due to the decrease in average pore size.

### 3.1. PFOA Filtration Studies

**Figure 2:**
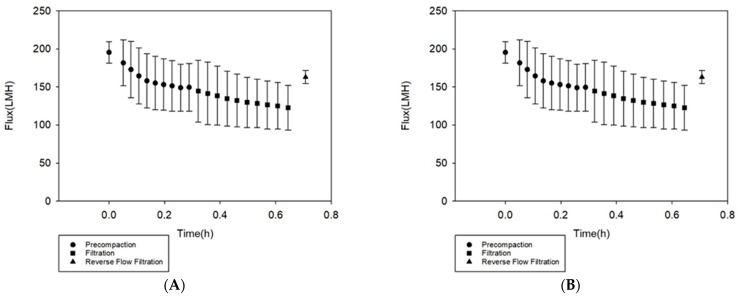
(**A**) Flux results (liters/m^2^-hr) for the phosphorene membrane at a constant pressure of 2.06 bar, showing the initial precompaction period, where pure water was filtered through the membranes until a near-constant flux value was attained. After precompaction, the filtration of PFOA was carried out, and it was followed by reverse flow filtration to simulate backwashing. (**B**) Normalized flux decline.

To study the permselectivity of the membranes towards PFOA, filtration studies were performed by filtering a 100 mg/L PFOA solution through the phosphorene membranes using crossflow filtration. The filtration flux profile is shown in Figure 2A, with all filtration experiments being performed at a pressure of 2.06 bar. From Figure 2A, during membrane precompaction, the initial and final pure water flux values of the membrane were 195 ± 14 LMH and 150 ± 31 LMH, respectively. At the end of precompaction, defined as once the pure water flux becomes constant, the PFOA filtration was started. The initial and final flux values for PFOA filtration were 145 ± 40 LMH and 123 ± 29 LMH. The flux after reverse flow filtration, used to simulate backwashing, was 163 ± 9 LMH; hence, the flux recovery was 84%. On average, total adsorption ratio of the membranes was 26%, the reversible resistance of the membrane was 10% and the irreversible resistance of the membrane was 16%. This indicates that PFOA only moderately adsorbed irreversibly to the membranes. The high flux recovery along with the low total adsorption ratio indicate a high anti-adsorption property [50]; therefore, the phosphorene membranes were able to successfully control PFOA adsorption. The standard deviations observed come from the variabilities that arise during the casting process. For example, the thickness of a membrane is partially responsible for the value of the flux through the membrane, with thicker membranes displaying lower flux values as compared to thinner membranes. While a doctor’s blade tool allows for setting of the desired thickness, spatial variations in laboratory-cast membranes are still common and possible [55]; hence, the high standard deviations. Flux declines were normalized to the initial flux for each duplicate filtration experiment (Figure 2B), and upon averaging those, the standard deviations decreased significantly supporting the notion that spatial variations associated with casting were responsible for the larger differences between runs.

Furthermore, PFOA was almost completely rejected by the membrane with a rejection of 99.9%. This excellent selectivity for PFOA with these membranes can be ascribed to two factors, size exclusion, based on pore size, and electrostatic repulsion between the membrane and the acid. The molecular weight of PFOA is 499 Da [56] or <0.14 μm [57], while previous studies have shown the average pore size of the phosphorene membranes was 0.0024 microns [46], so they were able to easily reject PFOA based on size exclusion. Furthermore, under neutral pH values, PFOA exists as the fully ionized component (COO^−^), so negatively charged [58], while under the same conditions, the membranes have also been previously shown to be negatively charged [46]. Therefore, the complete rejection of PFOA was further aided by electrostatic repulsion [59].

### 3.2. Hydrophobicity

**Figure 3:**
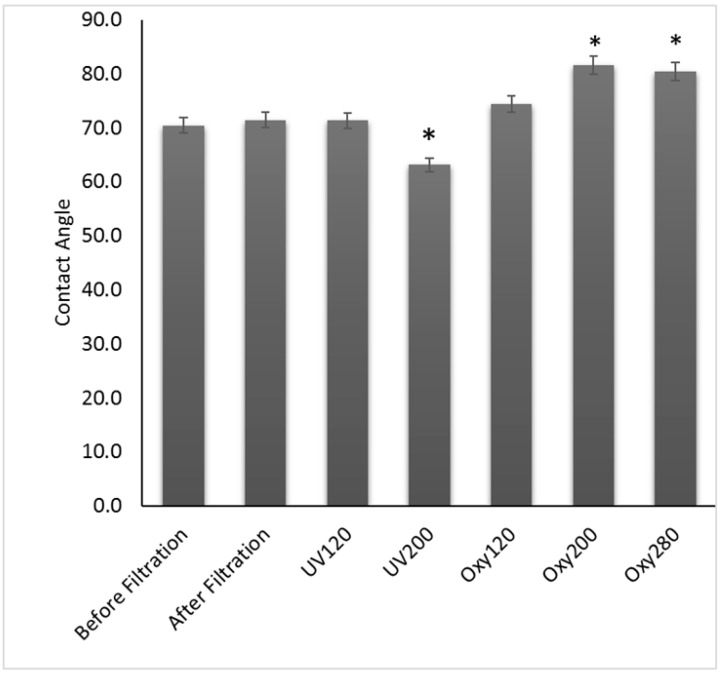
Water interaction parameter graph, to represent different levels of hydrophobicity, for the different membrane surface treatment techniques studied. The significant differences in contact angle between treatments with no treatment-alternative is indicated with * at *p* < 0.05.

Perfluoroalkyl chains can exhibit hydrophobic and hydrophilic tendencies because of the presence of fluorinated compounds, which introduce hydrophobicity, and carboxylic groups that introduce hydrophilicity [60]. The rigidly bound, non-bonding electron pairs that surround each fluorine atom in the C-F bonds, present in perfluoroalkyl compounds, are not easily polarized and thus prevent hydrogen bonding with polar and non-polar compounds. This increases with the degree of fluorine substitution at each carbon center and relies on the length of the perfluoroalkyl chain. Thus, longer perfluoroalkyl chains exhibit oleophobic properties, while shorter chains exhibit hydrophobic tendencies [61]. From Figure 3, the measured contact angle of the membrane before filtration was 70.4 ± 0.13°. After PFOA filtration, the contact angle did not change significantly, and it was 71.4 ± 0.76°. After irradiation with ultraviolet light for 120 min, the membranes did not experience changes in contact angle, 71.4 ± 2.47°, while with UV for 200 min, they became more hydrophilic with a contact angle of 63.1± 0.04°. The results of the one-way ANOVA with Tukey’s multiple comparisons test showed that the contact angle after UV irradiation for 120 was not different from the no-treatment alternative (*P* = 1.00), while UV for 200 min led to a significant decrease in contact angle (*P* = 0.008). This may be due to the formation of hydrophilic formic acid after the long-term photolysis reaction. Formic acid is one of major byproducts of PFOA photolysis by UV irradiation [62]. On the other hand, the membranes became more hydrophobic after catalytic oxygenation tested at 120, 200 and 280 min with average contact angles of 74.4 ± 2.51°, 81.6 ± 4.98°, and 80.4 ± 1.66°, respectively. The results of the one-way ANOVA with Tukey’s multiple comparisons test showed that oxygenation for 200 and 280 min led to significant increases in contact angle as compared to the no-treatment alternative (*P* = 0.002 and *P* = 0.005, respectively). This may be due to the breakdown of PFOA into smaller per fluorinated groups since these groups have stronger C-F bonds and are very hydrophobic.

### 3.3. Adsorbed PFOA Removal

**Figure 4:**
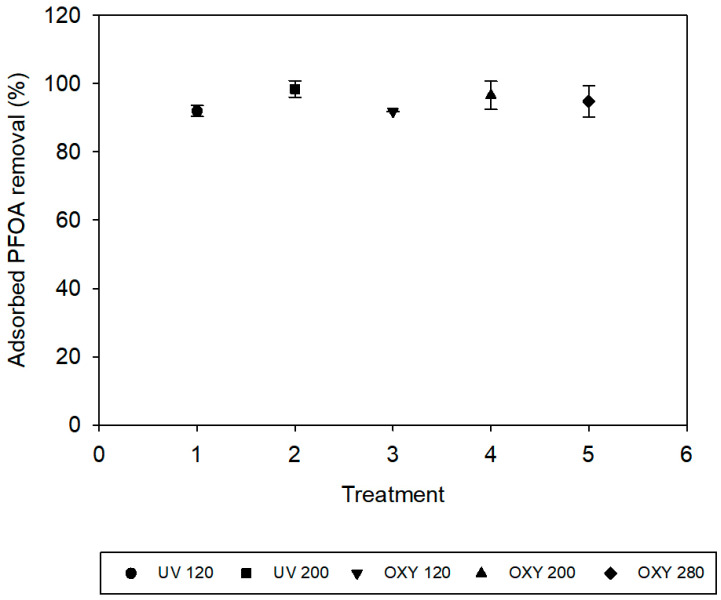
Removal of PFOA from the surface of the membrane after UV irradiation for two different periods of time, and after bubbling of oxygen for three different periods of time.

After PFOA was filtered through the membrane, the membranes were subjected to two treatment techniques to breakdown the PFOA adsorbed onto the membranes. Upon analysis of the permeates after each treatment for adsorbed PFOA, the PFOA removal is presented on Figure 4 as a function of the type of treatment used. Membranes that were subjected to UV irradiation for 120 min had a removal of 91.95 ± 1.6% of the PFOA that reversibly adsorbed the membranes. Membranes subjected to UV irradiation for 200 min had a removal of 98.4 ± 2.42%. Membranes subjected to oxygenation for 120 min displayed a removal of 91.8 ± 0.02%, while oxygenation for 200 min increased removal to 96.55 ± 4.1%. Lastly, oxygenation for 280 min showed a minor decrease in removal to 94.8 ± 4.59%. The results of the one-way ANOVA with Tukey’s multiple comparisons test also showed that the removal of adsorbed PFOA from the membrane surface was significantly increased in the UV treatment with duration of 200 min (*p* = 0.008). The similarity in the values of PFOA removal in these treatments suggests that both treatments, UV irradiation and oxygenation, were effective in removing the PFOA reversibly bound to the membranes. An underlying reason would be the photocatalytic properties of the membrane itself. Phosphorene-modified membranes have previously been observed to display tendencies for photocatalysis of organic compounds [46]; thus, it is proposed here that the PFOA was broken down into smaller compounds during treatment. These further buttresses the atomic profile scan results of the membrane surface and pores, where little to no fluorine molecules was detected in all the membranes. UV irradiation for 200 min had the highest adsorbed PFOA removal value because of phosphorene’s stronger light interaction with UV at 365 nm [46]. Furthermore, there was no fluoride detected, as measured by IC, in the reverse flow filtration samples from the membranes with no treatment or after they were exposed to UV and oxygenation. This suggests that, while PFOA is likely to break down into smaller chain compounds under treatments, the PFOA mineralization was not observed.

### 3.4. Membrane Morphology

**Figure 5:**
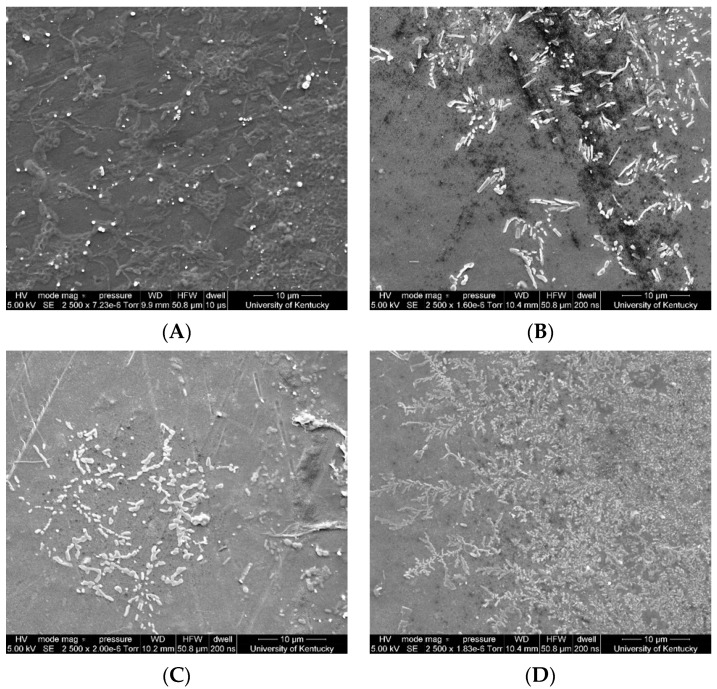
Surface SEM images of membranes after PFOA removal procedure: (**A**) baseline clean membrane, (**B**) after PFOA filtration, (**C**) after UV treatment of PFOA on membrane surface, and (**D**) after oxygen treatment of PFOA on membrane surface.

To further understand and visualize the effects of each treatment on the membrane PFOA adsorption, SEM surface images were taken after reverse flow filtration. Figure 5A shows the surface images of the clean membrane, the membrane after filtration of PFOA (Figure 5B), the membrane after filtration of PFOA and irradiation with UV for 200 min (Figure 5C), the membrane after filtration of PFOA and oxygenation for 280 min (Figure 5D). Organic matter adsorption is often characterized by the presence of a thick layer on the membrane surface [63]. Advanced oxidative processes can breakdown organic compounds by either altering their functional groups or dividing major aromatic moieties into smaller compounds, such as aliphatic organic acids [64]. From Figure 5, it was determined that membranes that were exposed to UV irradiation displayed the least PFOA adsorption, followed by the filtration alone membranes, and lastly membranes that were oxygenated. However, despite seeming to have the largest amount of PFOA of their surfaces, the adsorption on oxygenated membranes looked smaller in size and looser as compared all the other membranes. The presence of smaller compounds on the oxygenated membrane might be due to the generation of hydroxyl radicals during the treatment. Studies have shown that PFOA oxidation by hydroxyl radicals happens following a stepwise mechanism, where the cleavage of the carbon–carbon bond and the carboxylate group results in the generation of shorter chain perfluorinated groups [65], which also agrees with the observed increase in hydrophobicity (or contact angle) of the membrane after this treatment (Figure 3).

**Figure 6:**
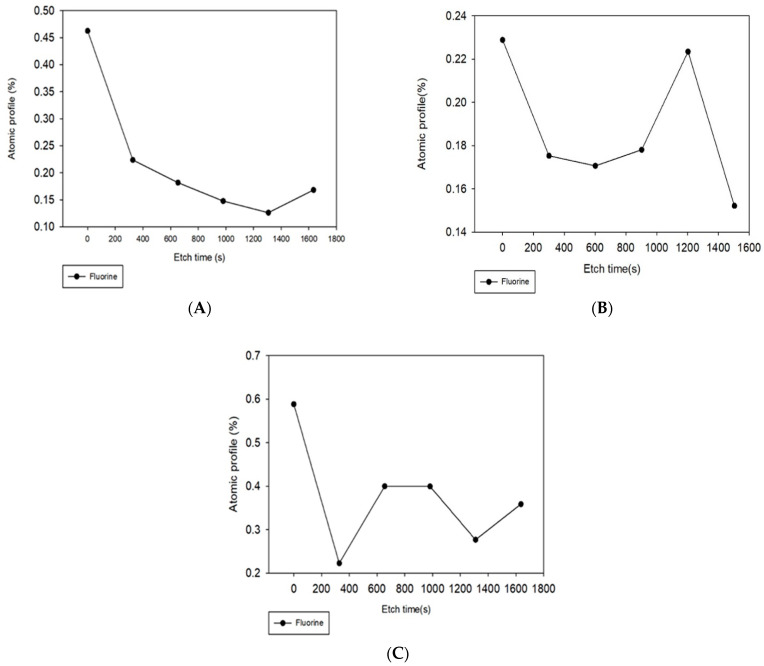
Depth atomic profile showing the percentage of fluorine on the membrane surfaces and within their pores after (**A**) filtration, (**B**) UV irradiation, and (**C**) oxygenation.

After each treatment, the chemical composition of the membrane surface and pores were studied via depth profile analysis using XPS. Figure 6 shows the elemental fluorine depth profile for the membranes after filtration of PFOA (Figure 6A), the membranes after filtration of PFOA and irradiation with UV for 200 min (Figure 6B), and the membranes after filtration of PFOA and oxygenation for 280 min (Figure 6C). In XPS depth profiling, the first point for all membranes, at 0 s etch time, shows the percentage of fluorine on the surface of the membrane, and subsequent values show the percentages as more of the membrane was etched; therefore, showing percentages in membrane pores. Other elements were carbon and oxygen; however, only fluorine is shown here since that is the element only present in PFOA, and not on the membranes; thus, this was the element tracked to monitor presence/removal of PFOA. Figure 6A, permeate after filtration, shows that all the fluorine was on the surface of the membranes with minimal amounts inside the pore structure. This directly agrees with the fact that the membrane pore sizes were smaller than the size of PFOA, and with Figure 5B that shows the high accumulation of PFOA on the membrane surface after filtration. It is important to note that the two treatments were not performed after reverse flow filtration but before it, so a direct comparison against Figure 6A is possible to show if removal occurred. Regarding UV treatment, Figure 6B shows that the amount of fluorine on the surface of the membranes was approximately half of that accumulated on the surface (Figure 6A), which indicates that UV irradiation was effective at the removal of fluorine from the membrane surface in agreement with Figure 5C. Furthermore, the presence of fluorine within the pores might indicate some destruction of PFOA into smaller compounds that were able to travel inside the membrane pores. On the other hand, oxygenation did not significantly impact the amount of fluorine present on the membrane surface as compared to reverse flow filtration and UV irradiation; however, the presence of fluorine inside the membrane pore structure suggests that some potential degradation of PFOA into smaller fluorine compounds might have occurred. Therefore, of all the treatment methods, the oxygenated membrane had the highest percentage of fluorine on its surface at 0.6%, followed by the membrane that was not treated after filtration at 0.5%, and lastly the UV irradiated membrane at 0.23%. This supports the contact angle findings (Figure 3) and the SEM images (Figure 5) that the oxygenated membranes had more perfluorinated groups present on their surfaces after the treatment.

**Figure 7:**
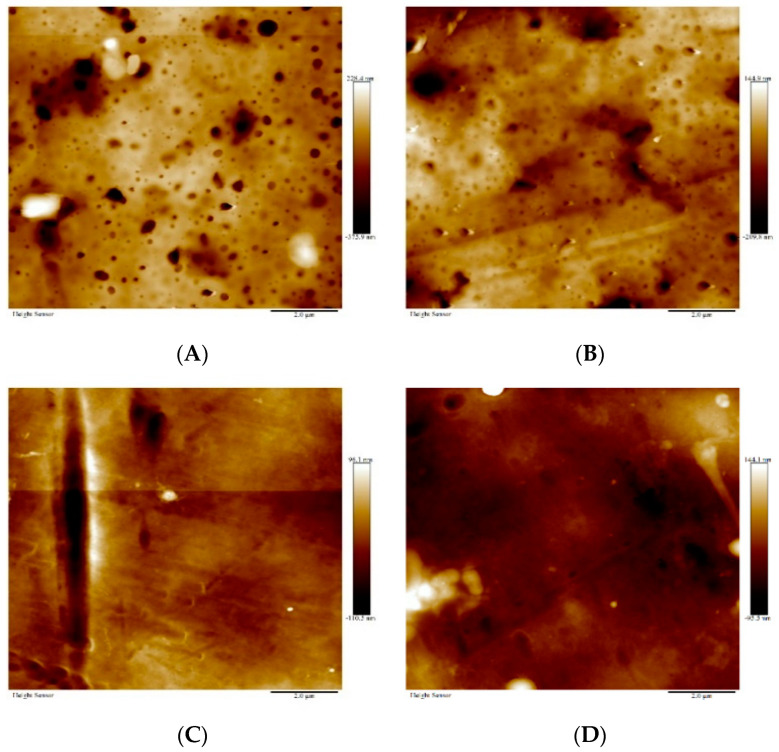
Atomic force microscope (AFM) images of membrane surface after treatment: (**A**) Plain/clean membrane, (**B**) the membrane after filtration of PFOA, (**C**) the membrane after filtration of PFOA and irradiation with UV, and (**D**) the membrane after filtration of PFOA and oxygenation.

AFM images of the membranes were taken to study the impact of the different treatments on the membrane surface roughness. Figure 7A–D shows the images of plain/clean membrane, the membrane after filtration of PFOA, the membrane after filtration of PFOA and irradiation with UV, the membrane after filtration of PFOA and oxygenation. The average root mean square values; that is, the surface roughness values, for each membrane were 73.7 ± 8.4 nm, 59.9 ± 13.7 nm, 26.03 ± 2.8 nm, and 35.8 ± 0.69 nm, respectively. The UV irradiated membranes were the smoothest, while the plain membranes were the roughest. From the SEM images of the membrane, it was observed that the UV membranes displayed the least PFOA adsorption, and this correlated with the roughness observed, as these were the smoothest membranes. The oxygenated membranes were the second smoothest membranes likely because of the degradation of the organic acid after this treatment. The particles were much smaller than the particles seen on the surface of the membrane after filtration and this may be why it had a lower roughness value than the filtration membrane.

### 3.5. Proposed Reaction Pathway Based on Literature Studies

**Figure 8:**
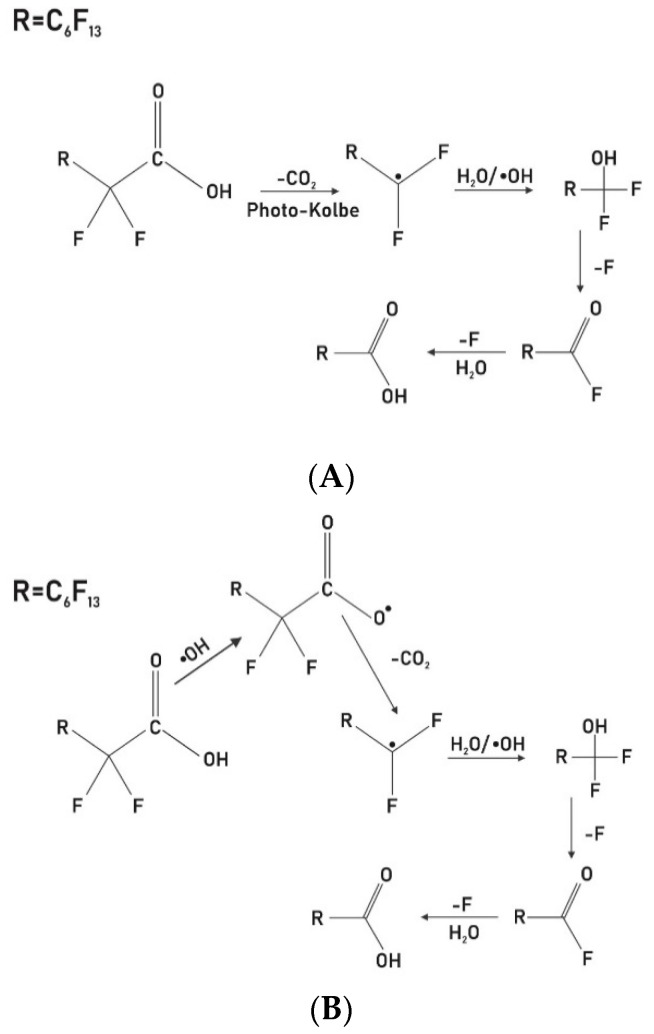
Possible pathways for the degradation of PFOA after (**A**) UV photocatalysis and (**B**) liquid aerobic oxidation [66].

Technologies that can potentially mineralize perflouororganics rather than transfer the fate of the contaminants from one phase to the other are highly desirable. The proposed degradation pathways after UV and aerobic oxidation treatments are shown in Figure 8A, pathway for the UV photocatalytic degradation of PFOA and Figure 8B, pathway for the liquid aerobic oxidation degradation of PFOA [66,67,68].

For the UV treatment, degradation is hypothesized to have been initiated by the scission of the C-C bonds in PFOA [66,67], as shown in Figure 8A. This reaction then potentially led to the formation of the perfluoro heptyl radicals. These radicals are further broken down to form an unstable perfluorinated alcohol intermediate (C_7_F_15_OH), which is quickly hydrolyzed to a perfluorinated carboxylic acid, C_6_F_13_COOH, a shorter-chained perfluorinated compound and the reaction continues until mineralization occurs [69]. Thus, perfluorooctanoic acid can be degraded via UV photocatalysis by a stepwise loss of a CF_2_ group [66]. The extent of the degradation was not determined in this study, and since there was no fluoride ions measured after reverse flow filtration, our data do not suggest that mineralization was achieved.

The combined system of photocatalyst and oxidation can produce a synergistic effect during the degradation of perfluoro organics [68]. The degradation process theoretically begins by the decarboxylation of PFOA to produce a perfluoro carboxylic radicals [66], which are broken down leading to the formation of perfluoro heptyl radicals [68] and then the cycle continues by a stepwise loss of a CF_2_ group until mineralization occurs (Figure 8B). The UV pathway is potentially shorter, which explains why a higher amount of adsorbed PFOA was removed, as seen in Figure 4, under the same time duration as the oxygenation treatment. This could be associated with phosphorene’s stronger interaction with UV as compared to visible light [46].

## 4. Conclusions

In this study, we demonstrated the successful removal of a persistent contaminant, perfluorooctanoic acid (PFOA) using nanohybrid membranes made of SPEEK and phosphorene. The membranes achieved nearly complete rejection of PFOA at 99%, and the flux recovery after reverse-flow filtration was 84%, indicating that PFOA did not significantly adsorb to the membranes. After filtration, the membranes were subjected to two treatments to destroy the PFOA that accumulated on the membrane surface. The first was UV photolysis that removed 98.4% of the adsorbed PFOA, while the second treatment was liquid aerobic oxygenation that led to a 96.6% removal. After treatment, the UV-treated membranes became smooth, hydrophilic and showed a minimal amount of fluorine left on the surface. Conversely, the oxygenated membranes became more hydrophobic and displayed a high amount of fluorine on the surface after treatment. The results from this study further confirms the photocatalytic characteristic of phosphorene. Given that PFOA is a persistent contaminant, this research has thus provided another avenue for the treatment of contaminated waters. This highlights the need for research into the scaleup of these dual functional membranes that exhibit very high rejection and removal of perfluorooctanoic acid.
